# Structure of the γ-d-glutamyl-l-diamino acid endopeptidase YkfC from *Bacillus cereus* in complex with l-Ala-γ-d-Glu: insights into substrate recognition by NlpC/P60 cysteine peptidases

**DOI:** 10.1107/S1744309110021214

**Published:** 2010-07-27

**Authors:** Qingping Xu, Polat Abdubek, Tamara Astakhova, Herbert L. Axelrod, Constantina Bakolitsa, Xiaohui Cai, Dennis Carlton, Connie Chen, Hsiu-Ju Chiu, Michelle Chiu, Thomas Clayton, Debanu Das, Marc C. Deller, Lian Duan, Kyle Ellrott, Carol L. Farr, Julie Feuerhelm, Joanna C. Grant, Anna Grzechnik, Gye Won Han, Lukasz Jaroszewski, Kevin K. Jin, Heath E. Klock, Mark W. Knuth, Piotr Kozbial, S. Sri Krishna, Abhinav Kumar, Winnie W. Lam, David Marciano, Mitchell D. Miller, Andrew T. Morse, Edward Nigoghossian, Amanda Nopakun, Linda Okach, Christina Puckett, Ron Reyes, Henry J. Tien, Christine B. Trame, Henry van den Bedem, Dana Weekes, Tiffany Wooten, Andrew Yeh, Keith O. Hodgson, John Wooley, Marc-André Elsliger, Ashley M. Deacon, Adam Godzik, Scott A. Lesley, Ian A. Wilson

**Affiliations:** a Stanford Synchrotron Radiation Lightsource, SLAC National Accelerator Laboratory, Menlo Park, CA, USA; b Joint Center for Structural Genomics, http://www.jcsg.org, USA; c Protein Sciences Department, Genomics Institute of the Novartis Research Foundation, San Diego, CA, USA; d Center for Research in Biological Systems, University of California, San Diego, La Jolla, CA, USA; e Program on Bioinformatics and Systems Biology, Sanford–Burnham Medical Research Institute, La Jolla, CA, USA; f Department of Molecular Biology, The Scripps Research Institute, La Jolla, CA, USA; g Photon Science, SLAC National Accelerator Laboratory, Menlo Park, CA, USA

**Keywords:** γ-d-glutamyl-l-diamino acid endopeptidase, cell-wall recycling, NlpC/P60, SH3b, cysteine peptidases, enzyme specificity

## Abstract

Dipeptidyl-peptidase VI from *Bacillus sphaericus* and YkfC from *Bacillus subtilis* have both previously been characterized as highly specific γ-d-glutamyl-l-­diamino acid endopeptidases. The crystal structure of a YkfC ortholog from *Bacillus cereus* (BcYkfC) at 1.8 Å resolution revealed that it contains two N-terminal bacterial SH3 (SH3b) domains in addition to the C-terminal catalytic NlpC/P60 domain that is ubiquitous in the very large family of cell-wall-related cysteine peptidases. A bound reaction product (l-Ala-γ-d-Glu) enabled the identification of conserved sequence and structural signatures for recognition of l-Ala and γ-d-Glu and, therefore, provides a clear framework for understanding the substrate specificity observed in dipeptidyl-peptidase VI, YkfC and other NlpC/P60 domains in general. The first SH3b domain plays an important role in defining substrate specificity by contributing to the formation of the active site, such that only murein peptides with a free N-terminal alanine are allowed. A conserved tyrosine in the SH3b domain of the YkfC subfamily is correlated with the presence of a conserved acidic residue in the NlpC/P60 domain and both residues interact with the free amine group of the alanine. This structural feature allows the definition of a subfamily of NlpC/P60 enzymes with the same N-terminal substrate requirements, including a previously characterized cyanobacterial l-­alanine-γ-d-glutamate endopeptidase that contains the two key components (an NlpC/P60 domain attached to an SH3b domain) for assembly of a YkfC-like active site.

## Abstract 2

The crystal structure of the highly specific γ-d-glutamyl-l-diamino acid endopeptidase YkfC from *Bacillus cereus* in complex with l-Ala-γ-d-Glu reveals the structural basis for the substrate specificity of NlpC/P60-family cysteine peptidases.

## 1. Introduction

**Figure 1:**
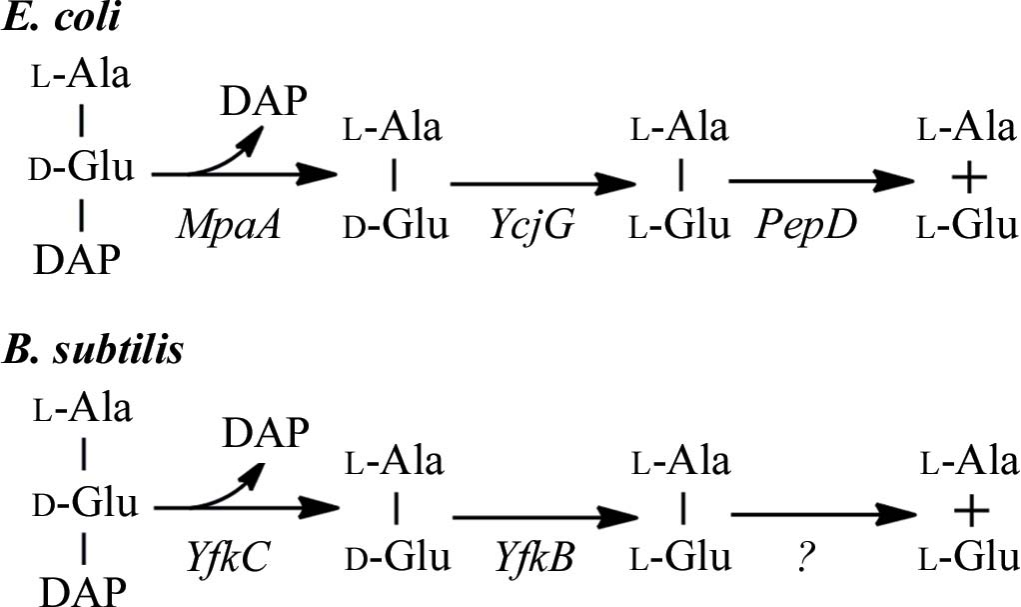
Proposed metabolic pathways for murein peptides in *E. coli* and *B. subtilis*.

Cell-wall turnover, an enzymatic process that results in the loss of peptidoglycan (PG) components, has been reported in many bacteria, including *Escherichia coli* and *Bacillus subtilis* (Doyle *et al.*, 1988 ▶). The products of the turnover are generally re-utilized through a process known as PG recycling (Park & Uehara, 2008 ▶). The molecular processes involved in cell-wall turnover and recycling are not currently well understood in comparison to cell-wall synthesis, particularly in bacteria other than *E. coli* (Park & Uehara, 2008 ▶; Uehara & Park, 2003 ▶, 2004 ▶, 2007 ▶; Uehara *et al.*, 2005 ▶). In *E. coli*, the cell wall is degraded by lytic transglycosylases that release anhydromuropeptides (GlcNAc-anhMurNAc-l-Ala-γ-d-Glu-DAP-d-Ala, where DAP is *meso*-diaminopimelic acid), which are imported into the cytoplasm, primarily by AmpG permease, and subsequently processed by *N*-acetyl-anhydromuramyl-l-alanine amidase (which cleaves between GlcNAc-anhMurNAc and l-Ala) and ld-carboxy­peptidase LdcA (which cleaves between DAP and d-Ala). Two fates are possible for the generated murein tripeptide l-Ala-γ-d-Glu-DAP. Under normal growth conditions, these tripeptides are recycled by Mpl ligase and returned to the peptidoglycan-biosynthetic pathway. During nutrient-limiting conditions, an additional pathway (Fig. 1 ▶) is likely to be involved in the murein tripeptide metabolism, as proposed in *E. coli* (Uehara & Park, 2003 ▶). MpaA endopeptidase, a metallocarboxypeptidase, specifically cleaves l-Ala-γ-d-Glu-DAP to produce l-Ala-γ-d-Glu and DAP. l-Ala-γ-d-Glu is then converted to l-Ala-l-Glu and subsequently to l-Ala and l-Glu by YcjG epimerase and PepD peptidase, respectively.

Some of the enzymes in the cell-wall recycling of *E. coli*, such as AmpG, AmpD, Mpl and MpaA, have no orthologs in *B. subtilis* (Park & Uehara, 2008 ▶), suggesting that the mechanism of cell-wall recycling may differ between the two bacteria. In *B. subtilis*, PG was proposed to be cleaved by a muramidase and an amidase to produce GlcNAc-MurNAc and stem peptides (Park & Uehara, 2008 ▶). The free peptides are imported into the cytoplasm by an unidentified permease and subsequently processed by YkfABC enzymes. YkfA, an ld-carboxy­peptidase, removes the terminal d-Ala. The generated tripeptide is further metabolized by a pathway that is functionally equivalent to that of *E. coli*, with YkfC as the γ-d-Glu-DAP endopeptidase and YkfB as the l-Ala-d-Glu epimerase (Fig. 1 ▶) (Schmidt *et al.*, 2001 ▶). Interestingly, while YkfB is homologous to YcjG of *E. coli*, YkfC is unrelated to MpaA in sequence and structure despite having an equivalent function.

YkfC contains a C-terminal NlpC/P60 cysteine peptidase domain. NlpC/P60 is a large family of cell-wall related cysteine peptidases that are broadly distributed in bacteria, viruses, archaea and eukaryotes (Anantharaman & Aravind, 2003 ▶; Bateman & Rawlings, 2003 ▶; Rigden *et al.*, 2003 ▶). Characterized NlpC/P60 enzymes are almost all γ-d-Glu-DAP (or γ-d-Glu-Lys) endopeptidases. While their bio­chemical function seems to be conserved, the physiological roles of NlpC/P60 proteins are diverse, including involvement in cell separation, expansion, differentiation, cell-wall turnover, cell lysis, protein secretion and virus infection (Smith *et al.*, 2000 ▶). Secreted NlpC/P60 proteins also have other roles in pathogenesis. The autolysin P60 of *Listeria monocytogenes* is involved in host-cell invasion (Kuhn & Goebel, 1989 ▶), enterotoxin FM of *B. cereus* in food poisoning (Asano *et al.*, 1997 ▶), and SagA of *Enterococcus faecium* is a secreted antigen that binds to extracellular matrix proteins (Teng *et al.*, 2003 ▶).

NlpC/P60 proteins can be lethal to bacteria owing to their ability to compromise cell-wall integrity or cell-wall biosynthesis. Therefore, their activities are tightly controlled through multiple mechanisms (Smith *et al.*, 2000 ▶); their expression is regulated at the transcription level and their cellular localization is dependent on their physio­logical roles. Furthermore, their atomic structures are highly optimized to precisely define their substrate specificity (Xu *et al.*, 2009 ▶). NlpC/P60 proteins are often fused to auxiliary domains, many of which are known cell-wall binding modules (*e.g.* LysM and the choline-binding domain). Thus, it is generally assumed that these auxiliary domains function as targeting domains which localize their proteins to the cell wall. The functional synergy between the NlpC/P60 domains and their auxiliary domains is currently not fully understood. We have previously determined the crystal structure of a γ-d-Glu-DAP endopeptidase from cyanobacteria (AvPCP/NpPCP; *Anabaena variabilis*/*Nostoc punctiforme* PG cysteine peptidase; Xu *et al.*, 2009 ▶) and showed that it contained an N-terminal bacterial SH3 (SH3b) domain and a C-terminal NlpC/P60 domain. We proposed that the SH3b domain of this enzyme is important in defining the substrate specificity of the peptidase domain. However, the mechanism of substrate recognition by NlpC/P60 and SH3b was not firmly established. Here, we report the crystal structure of YkfC from *B. cereus* (BcYkfC) in complex with l-Ala-γ-d-Glu. BcYkfC shares 40% sequence identity with YkfC from *B. subtilis*, which has previously been biochemically characterized (Schmidt *et al.*, 2001 ▶). Thus, we now have the first detailed view of substrate recognition by an NlpC/P60 protein.

## 2. Material and methods

### 2.1. Sequence analysis

Homologs of BcYkfC were identified using *PSI-BLAST* (Altschul *et al.*, 1997 ▶; three iterations) against the nonredundant (nr) protein sequence database at the National Center for Biotechnology Information (NCBI) using the sequence of the catalytic domain of BcYkfC as the probe (residues 210–333). An alignment length of ≥70 and an *E* value of ≤0.02 were used to extract a subset of hits. These proteins (2599 sequences) were aligned using *HMMALIGN* (Eddy, 1998 ▶) against the default global alignment profile of NlpC/P60 domains (PF00877) from the PFAM database v.23 (Bateman *et al.*, 2004 ▶). YkfC-subfamily candidates were extracted from the above aligned subset based on the presence of an aspartate corresponding to position 256 of BcYkfC. The full-length sequences were then aligned and clustered using *PIPEALIGN* (Plewniak *et al.*, 2003 ▶). Only sequences that also contained a conserved tyrosine corresponding to position 118 of BcYkfC were classified into the YkfC subfamily. Plots of sequence conservation in the active site were prepared using *WEBLOGO* (Crooks *et al.*, 2004 ▶).

### 2.2. Protein expression and purification

Clones were generated using the Polymerase Incomplete Primer Extension (PIPE) cloning method (Klock *et al.*, 2008 ▶). The gene encoding BcYkfC (GenBank NP_979181; Swiss-Prot Q736M3) was amplified by polymerase chain reaction (PCR) from *B. cereus* NRS248 ATCC 10987 genomic DNA using *PfuTurbo* DNA polymerase (Stratagene) and I-PIPE (Insert) primers (forward primer, 5′-ctgtacttccagggcGAAGAGAAGAAAGATAGTAAGGCGT-3′; reverse primer, 5′-aattaagtcgcgttaAGGTAAGTAACGACGCGCAC­CAGCG-3′; target sequence in upper case) that included sequences for the predicted 5′ and 3′ ends. The expression vector pSpeedET, which encodes an amino-terminal tobacco etch virus (TEV) protease-cleavable expression and purification tag (MGSDKIHHHHHHENLYFQ/G), was PCR-amplified with V-PIPE (Vector) primers (forward primer, 5′-taacgcgacttaattaactcgtttaaacggtctccagc-3′; reverse primer, 5′-gccctggaagtacaggttttcgtgatgatgatgatgatg-3′). V-PIPE and I-PIPE PCR products were mixed to anneal the amplified DNA fragments together. *E. coli* GeneHogs (Invitrogen) competent cells were transformed with the I-PIPE/V-PIPE mixture and dispensed onto selective LB–agar plates. The cloning junctions were confirmed by DNA sequencing. Using the PIPE method, the gene segment encoding residues Met1–Ala23 was omitted as these residues were predicted to form a signal peptide. Expression was performed in a selenomethionine-containing medium with suppression of normal methionine synthesis. At the end of fermentation, lysozyme was added to the culture to a final concentration of 250 µg ml^−1^ and the cells were harvested and frozen. After one freeze–thaw cycle, the cells were sonicated in lysis buffer [50 m*M* HEPES pH 8.0, 50 m*M* NaCl, 10 m*M* imidazole, 1 m*M* tris(2-carboxyethyl)phosphine–HCl (TCEP)] and the lysate was clarified by centrifugation at 32 500*g* for 30 min. The soluble fraction was passed over nickel-chelating resin (GE Healthcare) pre-equilibrated with lysis buffer, the resin was washed with wash buffer [50 m*M* HEPES pH 8.0, 300 m*M* NaCl, 40 m*M* imidazole, 10%(*v*/*v*) glycerol, 1 m*M* TCEP] and the protein was eluted with elution buffer [20 m*M* HEPES pH 8.0, 300 m*M* imidazole, 10%(*v*/*v*) glycerol, 1 m*M* TCEP]. The eluate was buffer-exchanged with TEV buffer (20 m*M* HEPES pH 8.0, 200 m*M* NaCl, 40 m*M* imidazole, 1 m*M* TCEP) using a PD-10 column (GE Healthcare) and incubated with 1 mg TEV protease per 15 mg of eluted protein. The protease-treated eluate was run over nickel-chelating resin (GE Healthcare) pre-equilibrated with HEPES crystallization buffer (20 m*M* HEPES pH 8.0, 200 m*M* NaCl, 40 m*M* imidazole, 1 m*M* TCEP) and the resin was washed with the same buffer. The flowthrough and wash fractions were combined and concentrated to 18.8 mg ml^−1^ as determined using the Coomassie Plus Protein Assay Reagent (Pierce) by centrifugal ultrafiltration (Millipore) for crystallization trials. The oligomeric state of BcYkfC was determined using a 0.8 × 30 cm Shodex Protein KW-803 column (Thomson Instruments) pre-calibrated with gel-filtration standards (Bio-Rad).

### 2.3. Crystallization

BcYkfC was crystallized by mixing 200 nl protein solution with 200 nl crystallization solution and equilibrating against a 50 µl reservoir solution using the nanodroplet vapor-diffusion method (Santarsiero *et al.*, 2002 ▶) with standard Joint Center for Structural Genomics (JCSG; http://www.jcsg.org) crystallization protocols (Lesley *et al.*, 2002 ▶). The crystallization solution was composed of 0.2 *M* sodium chloride, 50%(*v*/*v*) PEG 200 and 0.1 *M* phosphate–citrate pH 4.2. A needle-shaped crystal of approximate dimensions 100 × 15 × 15 µm was harvested after 29 d at 277 K. No additional cryoprotectant was added to the crystal. Initial screening for diffraction was carried out using the Stanford Automated Mounting system (SAM; Cohen *et al.*, 2002 ▶) at the Stanford Synchrotron Radiation Lightsource (SSRL, Menlo Park, California, USA).

### 2.4. Data collection, structure solution and refinement

Multi-wavelength anomalous diffraction (MAD) data were collected at wavelengths corresponding to the peak, high-energy remote and inflection wavelengths of a selenium MAD experiment at 100 K using a MAR CCD 325 detector (Rayonix) on SSRL beamline 11-1. Processing of the diffraction data and initial structure solution were carried out using the automatic structure-solution script *autoXDSp* developed at the JCSG (unpublished work). This script shepherds the structure-determination process, as summarized below, using preset rules through a decision-tree that mimics that used by an experienced crystallographer. The calculations are parallelized on a computer cluster such that initial maps and models can usually be obtained within 1 h of the completion of data collection. In summary, the MAD data were integrated and reduced using *XDS* and then scaled with the program *XSCALE* (Kabsch, 1993 ▶, 2010 ▶). Selenium sites were located with *SHELXD* (Sheldrick, 2008 ▶). Phase refinement and automatic model building were performed using *autoSHARP* (Bricogne *et al.*, 2003 ▶) and *ARP*/*wARP* (Cohen *et al.*, 2004 ▶). This automated process produced an initial model that was 92% complete. Further model completion and refinement were performed manually with *Coot* (Emsley & Cowtan, 2004 ▶) and *REFMAC* (Murshudov *et al.*, 1997 ▶) from the *CCP*4 suite (Collaborative Computational Project, Number 4, 1994 ▶). Data and refinement statistics are summarized in Table 1 ▶. Analysis of the stereochemical quality of the model was accomplished using *MolProbity* (Chen *et al.*, 2010 ▶). All molecular graphics were prepared with *PyMOL* (DeLano Scientific) unless specifically stated otherwise. Atomic coordinates and experimental structure factors for BcYkfC at 1.8 Å resolution have been deposited in the PDB with accession code 3h41.

### 2.5. Molecular modeling

Molecular docking was performed using the same protocol as described previously (Xu *et al.*, 2009 ▶) using *Glide* v.5.0 (Schrödinger LLC). The positions of the bound ligand were used as restraints such that the l-Ala-γ-d-Glu portion of the docked substrate adopted a similar conformation as seen in the crystal structure. In order to perform the docking studies, the tri-oxidized cysteine (OCS238) in the crystal structure was replaced with the reduced form, which is needed for reaction in the papain family of cysteine peptidases. Furthermore, only the side-chain conformer with higher occupancy in the crystal structure was considered in the docking experiments when multiple conformations were observed for an active-site residue.

## 3. Results and discussion

### 3.1. Genomic context

Full-length BcYkfC (strain *B. cereus* ATCC 10987) contains 333 residues (molecular weight 37.3 kDa), the first 23 of which are pre­dicted to be a signal peptide by the *Phobius* web server (Kall *et al.*, 2007 ▶). Despite being homologous to BcYkfC, *B. subtilis* YkfC (296 residues) does not contain a signal peptide, suggesting that the two proteins function at different cellular locations.

**Figure 2:**
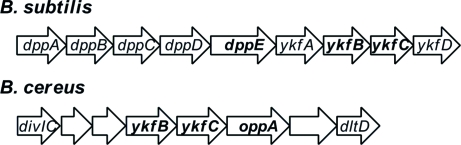
Genomic context of the *ykfB* and *ykfC* genes in *B. subtilis* and *B. cereus*.

As in *B. subtilis*, the *ykfC* gene (BCE_2878) is adjacent to the *ykfB* l-Ala-d-Glu epimerase gene (BCE_2879) in the *B. cereus* genome. The YkfB epimerases (sequence identity 50%) from both bacteria do not contain predicted signal peptides. The genome association of *ykfB* and *ykfC* is also observed in other bacteria (*e.g. Listeria innocua*, *Acidobacterium* sp., *Solibacter usitatus*, *Bacteroides thetaiota­omicron* and *Gramella forsetii*). However, the genome context of *ykfBC* is different in *B. subtilis* compared with *B. cereus* (Fig. 2 ▶). The *YkfA–D* genes of *B. subtilis* are located next to the *dppB–E* dipeptide ABC transporter operon, whereas the *YkfBC* genes of *B. cereus* are located downstream of *divIC*, which encodes a putative cell-division protein. Downstream of *ykfC* is an *oppA* gene that is homologous to *dppE* (34% sequence identity). Both genes are predicted to encode periplasmic dipeptide-binding proteins.

Based on the presence/absence of the signal peptide on YkfC and YkfB, as well as their genomic contexts, we suggest that the strategy for metabolizing murein peptides is likely to differ between *B. subtilis* and *B. cereus*. In *B. cereus*, the murein peptides are likely to be broken down outside the cell, with the resulting dipeptide l-Ala-γ-d-Glu being imported into cytoplasm for further processing by YkfB, while the reactions catalyzed by both YkfC and YkfB are likely to occur in the cytoplasm in *B. subtilis*.

### 3.2. Structure determination and quality of the model

The crystal structure of BcYkfC was determined using the high-throughput structural genomics pipeline implemented at the JCSG (Lesley *et al.*, 2002 ▶). The selenomethionine derivative of BcYkfC was expressed in *E. coli* with an N-terminal TEV-cleavable His tag and purified by metal-affinity chromatography. In order to improve the chance of obtaining crystals, the predicted N-terminal signal peptide (residues 1–23) was not included in the cloned construct. The data were indexed in space group *C*2 and the structure was determined at 1.79 Å resolution with one molecule per asymmetric unit using the MAD method (*R*_cryst_ = 16.3%, *R*_free_ = 19.7%). The electron density for the main chain was well defined throughout the entire molecule. The mean residual error of the coordinates was estimated to be 0.11 Å by the diffraction-component precision index (DPI) method (Cruickshank, 1999 ▶). The model of BcYkfC displays good geometry, with an all-atom clash score of 5.07, and the Ramachandran plot produced by *MolProbity* (Chen *et al.*, 2010 ▶) shows that all residues, but one, are in allowed regions, with 97.7% in favored regions. Only one residue is flagged as a rotamer outlier. The Ramachandran (Pro305) and rotamer (His303) outliers are supported by well defined electron density. Since these residues are either close to or part of the active site, these structural deviations from ideality are likely to be of functional relevance. Additionally, two *cis*-peptides (Asn57–Pro58 and Asn154–Pro155) are also supported by clear electron density. The final model of BcYkfC contains residues 29–333, one dipeptide l-Ala-γ-d-Glu, one phosphate, six polyethylene glycol (PEG) fragments from the crystallization solution and 265 waters. The residual residue (Gly0) from the cleaved N-terminal purification tag, residues 24–28 and the side chains of Glu201 and Arg270 were disordered and were not included in the final model. Data-collection, refinement and model statistics are summarized in Table 1 ▶.

### 3.3. Overall structure

**Figure 3:**
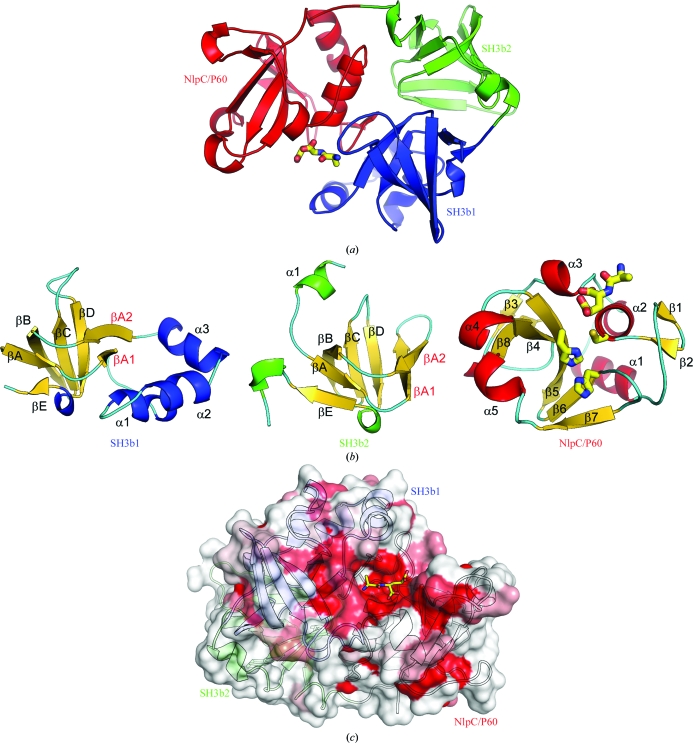
Crystal structure of YkfC from *B. cereus* in complex with l-Ala-γ-d-Glu. (*a*) Ribbon representation of BcYkfC, highlighting its domain organization. SH3b1 is depicted in blue, SH3b2 in green and NlpC/P60 in red. The bound l-Ala-γ-d-Glu is shown as a stick model. (*b*) Ribbon representations of individual domains, showing the secondary-structure elements. (*c*) Molecular surface of YkfC colored by sequence conservation. The surface color gradient indicates the level of sequence conservation from the most conserved residues (deep red) to nonconserved residues (white).

BcYkfC is likely to be a monomer in solution, as supported by crystal-packing analysis and analytical size-exclusion chromatography. The structure of BcYkfC consists of three domains: two SH3b domains (SH3b1, residues 29–129; SH3b2, residues 130–207) and a C-terminal NlpC/P60 cysteine peptidase domain (residues 208–333) (Figs. 3 ▶ *a* and 3 ▶ *b*). The two SH3b domains are similar to each other, with an r.m.s.d. of 2.2 Å for 57 aligned C^α^ atoms (sequence identity of 13%). A structural similarity search using *DALI* (Holm & Sander, 1995 ▶) did not find any other structures with the same three-domain architecture. However, a number of significantly similar substructures were identified and are summarized in Table 2 ▶. The SH3b domains of BcYkfC are similar to other SH3b domains (Holm & Sander, 1995 ▶), including the N-­terminal domain of cyanobacterial γ-d-Glu-DAP endopeptidases (Xu *et al.*, 2009 ▶), the GW domains of internalin B (Marino *et al.*, 2002 ▶), the cell-wall-targeting domain of glycylglycine endopeptidase ALE-1 (Lu *et al.*, 2006 ▶), PhnA-like protein (Srisailam *et al.*, 2006 ▶) and endolysin PlyPSA (Korndorfer *et al.*, 2006 ▶), as well as many eukaryotic SH3 domains. A β-hairpin within the so-called ‘RT loop’ (*i.e.* the loop between βA and βB) region appears to be a common and unique structural feature of SH3b domains compared with their eukaryotic counterparts (Xu *et al.*, 2009 ▶). The BcYkfC SH3b domains both contain this conserved structural motif (βA1–βA2). However, SH3b1 contains a novel helical insertion (α1–α3) that is not seen in previous SH3b structures or in SH3b2 (Fig. 3 ▶ *b*). Prokaryotic SH3-like domains have also been implicated in polypeptide binding (Wylie *et al.*, 2005 ▶) and metal binding (Pohl *et al.*, 1999 ▶). Although these SH3-like domains contain a similar five-stranded (βA–βE) core, they display much larger structural differences (and are not among significant *DALI* hits with *Z* > 2.0) and lack the β-­hairpin in the RT-loop region when compared with SH3b domains. The C-terminal NlpC/P60 catalytic domain of BcYkfC is remotely related to the papain family of cysteine peptidases (Anantharaman & Aravind, 2003 ▶), with highest similarity to cyanobacterial γ-d-Glu-DAP endopeptidases (Xu *et al.*, 2009 ▶), *E. coli* lipoprotein Spr (Aramini *et al.*, 2008 ▶) and two uncharacterized proteins (PDB codes 2p1g and 2im9; NYSGXRC, unpublished work) (Table 2 ▶).

The three domains of BcYkfC are arranged in a triangle such that each domain interacts with the two other domains. The interface (∼570 Å^2^ per domain) between the two SH3b domains is mostly hydrophobic and is centered on interactions between the βA–βA1 and βA2–βB loops of SH3b1 and the βA2 and βB strands and βD–βE loop of SH3b2. The SH3b1–NlpC/P60 domain interface (903 Å^2^ buried surface per domain) is mediated through α2–α3–βA1 and the βC–βD loop of SH3b1, and α1–α2–α3 and the β6–β7 loop of NlpC/P60. The active site is located at this SH3b1–NlpC/P60 interface, whereas the SH3b2 domain is distal to the active site. A multiple sequence alignment of full-length homologs of YkfC (37 sequences; average sequence identity of 54%) indicates that most of the highly conserved residues are either buried inside the protein or clustered around the active site (Fig. 3 ▶ *c*).

### 3.4. Active site

**Figure 4:**
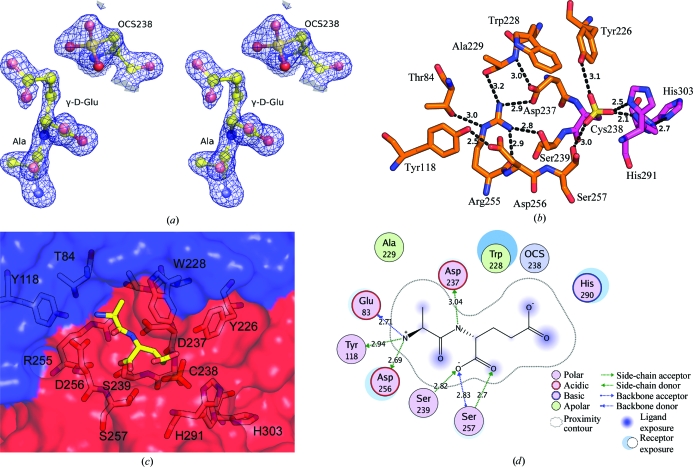
Active site and recognition of l-Ala-γ-d-Glu by BcYkfC. (*a*) Stereoview of a 2*F*_o_ − *F*_c_ OMIT map, where l-Ala-γ-d-Glu and OCS238 were omitted from phasing/refinement, contoured at 1.5σ. (*b*) The extensive hydrogen-bond network in the active site of BcYkfC. Hydrogen bonds and distances are shown as dashed lines. (*c*) l-Ala-γ-d-Glu (stick representation; yellow C atoms) is located in the active site at the interface of the SH3b1 domain (blue) and the NlpC/P60 domain (red). (*d*) The interaction between l-Ala-γ-d-Glu and the active site of YkfC. This figure was generated using the program *MOE* 2008.10 (Chemical Computing Group Inc.).

The catalytic triad of the peptidase domain consists of Cys238, His291 and His303. The catalytic Cys238 is oxidized (OCS) in the crystal based on the electron density (Fig. 4 ▶ *a*). Since an oxidized cysteine can no longer function as a nucleophile in the reaction (Storer & Ménard, 1994 ▶), the enzyme in the crystal is inactive. The con­formation of the cysteine side chain is not significantly affected by the oxidation as its side chain is in a similar location and conformation as in other NlpC/P60 structures (Xu *et al.*, 2009 ▶).

The dipeptide l-Ala-γ-d-Glu was identified from well defined electron density in the active site of BcYkfC (Fig. 4 ▶ *a*). As the dipeptide was not added during protein purification or crystallization, it was most likely to have been obtained during protein expression in *E. coli*. Since this dipeptide is one of the reaction products of BcYkfC (Fig. 2 ▶), it unequivocally identifies the S2–S1 binding-site cavity, which is formed by residues from both the SH3b1 (Glu83, Thr84 and Tyr118) and NlpC/P60 domains (Tyr226, Trp228, Ala229, Asp237, Arg255, Asp256, Ser257, His290 and His291). Two charged residues, Asp237 and Arg255, which are highly conserved in NlpC/P60 domains, are involved in a hydrogen-bond network that connects many residues in the active site (Fig. 4 ▶ *b*).

Two active-site residues, Ser257 and His291, display two discrete side-chain conformations (Fig. 4 ▶ *b*). The first conformer of His291 (occupancy modeled as 0.7), which facilitates hydrogen bonding to His303, is identical to the corresponding histidine of the catalytic dyad in papain and other NlpC/P60 enzymes. The second rotamer points His291 towards the solvent. The side-chain isomerism in the active site is likely to be a consequence of the observed oxidized state of the catalytic Cys238, since the oxidized Cys238 makes multiple hydrogen-bond interactions with nearby side chains, including Tyr226, Ser257 and His291 (Fig. 4 ▶ *b*).

### 3.5. Recognition of l-Ala-γ-d-Glu

The active-site residues form a pocket that is highly complementary in shape and chemical properties to the ligand (Figs. 4 ▶ *c* and 4 ▶ *d*). The average *B* value for the bound ligand is 26 Å^2^ (the overall *B* value of the protein is 18 Å^2^), indicating that it is well ordered with almost full occupancy in the active site. The interface between the dipeptide and the protein buries a total surface area of 490 Å^2^. The dipeptide is stabilized by multiple hydrogen bonds. The free amine of l-Ala in the S2 pocket makes hydrogen bonds to Glu83 O and the side chains of Tyr118 (OH) and Asp256 (O^δ1^), which are highly conserved in the YkfC subfamily of NlpC/P60 enzymes (see below). The α-NH group of γ-d-Glu in the S1 site forms a weak hydrogen bond to Asp237 O^δ2^. The α-carboxyl of d-Glu is stabilized by hydrogen-bonding interactions with Ser239 and Ser257 (Fig. 4 ▶ *d*). On the solvent-exposed side of the substrate, waters are also involved in the hydrogen-bond network with the substrate and the enzyme (not shown). The l-Ala methyl side chain points towards a hydrophobic pocket defined by the side chains of Trp228 and Ala229. Additionally, the aliphatic C atoms of d-Glu are involved in hydrophobic contacts with Trp228. As a result, Trp228 contributes to both the S2 and S1 binding sites.

### 3.6. YkfC is specific for murein peptides with free N-terminal l-Ala

**Figure 5:**
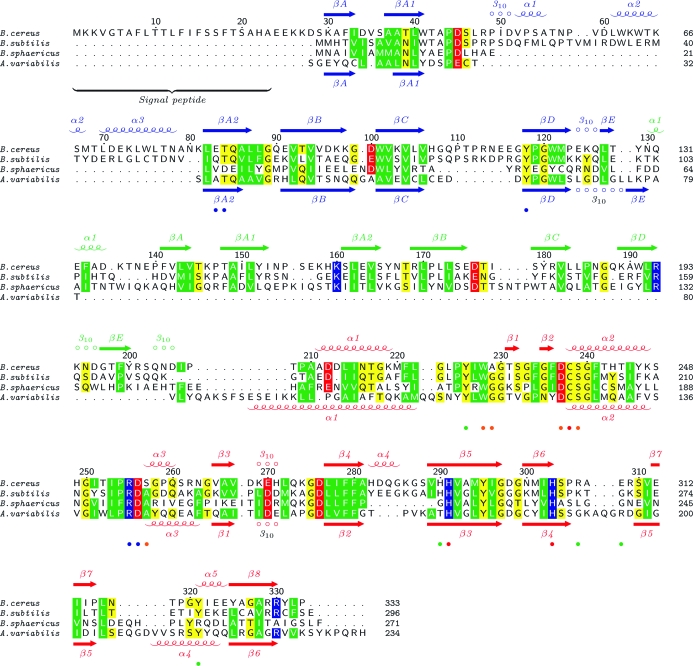
Sequence alignment of YkfC from *B. subtilis* and *B. cereus*, dipeptidyl-peptidase VI (DPP VI) from *B. sphaericus* and a γ-d-glutamyl-l-diamino acid endopeptidase from *A. variabilis* (AvPCP). The sequence numbering and secondary-structure elements of YkfC from *B. cereus* and AvPCP are indicated at the top and bottom, respectively. The alignment was generated by merging and manually editing the structure-based sequence alignment of BcYkfC and AvPCP with the sequence alignment of the top three sequences. The active-site residues are marked with colored dots at the bottom (blue, S2; orange, S1; red, catalytic triad; green, potential S′ sites).

The active site of *B. subtilis* YkfC is highly conserved compared with that of BcYkfC (Fig. 5 ▶). Dipeptidyl peptidase VI (DPP VI) from *B. sphaericus* is a γ-d-Glu-DAP(Lys) dipeptidase that is found in the cytoplasm during sporulation (Vacheron *et al.*, 1979 ▶). DPP VI has strict specificity for murein peptides with an N-terminal l-Ala. The crystal structure of BcYkfC provides a structural basis for this specificity. DPP VI and BcYkfC have only 21% sequence identity, but the same sets of key residues are conserved, as expected given their similar folds and function (Fig. 5 ▶). Thus, DPP VI is clearly a homolog of BcYkfC, except that its SH3b1 domain has no large helical insertion (α1–α3) between βA1 and βA2 and it has a shorter βC–βD loop (Fig. 5 ▶). Furthermore, the S2 and S1 binding sites for l-Ala-γ-d-Glu are highly conserved between DPP VI and BcYkfC, including the conserved tyrosine (Tyr118 of YkfC), which is the only residue from SH3b1 whose side chain interacts with l-Ala. The residues from the catalytic domain that contribute to the S2 and S1 sites are identical in BcYkfC and DPP VI (except for an Ala/Ser mutation at position 257 of BcYkfC). Therefore, we conclude that both the *B. subtilis* and the *B. cereus* YkfCs are likely to have very similar substrate specificity for murein peptides that contain an N-terminal l-Ala. Enzymes with this specificity cannot cleave PG-biosynthesis precursors such as UDP-MurNAc-pentapeptide and thus are not likely to interfere with the PG-synthesis pathway.

### 3.7. S′ sites of YkfC and docking studies

**Figure 6:**
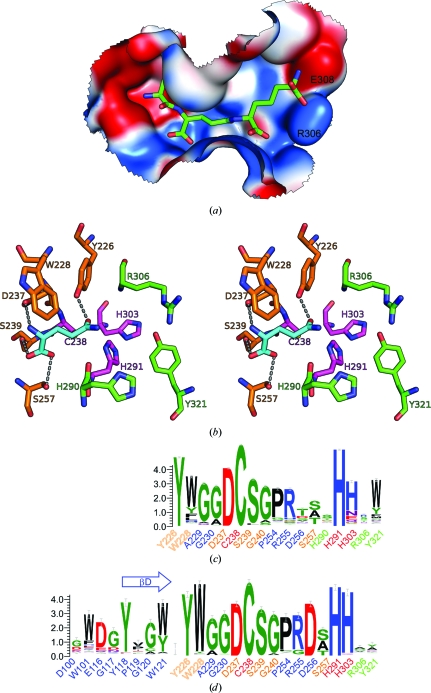
Models of substrate recognition by BcYkfC. (*a*) l-Ala-γ-d-Glu-DAP was docked into the active site of BcYkfC. The protein surface is colored according to a gradient in electrostatic potential from negative (red) to positive (blue) (*MOE* 2008.10; Chemical Computing Group Inc.). (*b*) Stereoview of the specific interactions (four polar, one nonpolar) of γ-d-Glu (cyan) in the context of the tripeptide by five residues of YkfC (Tyr226, Trp228, Asp237, Ser239 and Ser257). The protein residues are colored according to subsite (S1, orange; catalytic triad, magenta; S1′, green). (*c*) Sequence conservation of the active sites in NlpC/P60 domains based on 2277 NlpC/P60 domains with an intact catalytic dyad (Cys238 and His291) and a conserved Tyr226 (blue, S2; orange, S1; red, catalytic triad; green, S′ sites). (*d*) Sequence conservation of the active sites in the YkfC subfamily of NlpC/P60 enzymes based on 282 sequences selected based on the presence of a conserved aspartate residue at position 256 of BcYkfC. The conservation of this residue is highly correlated with a conserved tyrosine in the βD region of the SH3b domain (Tyr118 of BcYkfC); both of these residues interact with the free amine of l-Ala of the substrate.

*B. subtilis* YkfC has previously been shown to process the tri­peptide l-Ala-γ-d-Glu-l-Lys, the tetrapeptide l-Ala-γ-d-Glu-l-Lys-d-­Ala and the pentapeptide l-Ala-γ-d-Glu-l-Lys-d-Ala-d-Ala (Schmidt *et al.*, 2001 ▶). DPP VI also displayed no particular specificity towards the additional residues attached after DAP (or Lys; Vacheron *et al.*, 1979 ▶). Residues in BcYkfC that potentially form the S′ sites include His290, His291, Arg306, Glu308, Arg309, Tyr321 and Glu324. These residues are generally less conserved among close BcYkfC homologs (Fig. 5 ▶), suggesting that the specificity of YkfC is determined primarily at the S2 and S1 sites as described above. The catalytic mechanism of NlpC/P60 is currently unknown, but it is likely to be similar to that of papain based on the similar arrangement of their catalytic residues (Anantharaman & Aravind, 2003 ▶; Aramini *et al.*, 2008 ▶; Xu *et al.*, 2009 ▶), except for two interesting variations of important catalytic residues. The third polar residue of the catalytic triad in NlpC/P60 is more often a histidine rather than the asparagine in papain (Bateman *et al.*, 2004 ▶). A tyrosine (Tyr226 of BcYkfC) is located at the position equivalent to Gln119 in papain that is thought to be important for catalysis by stabilizing the transition state (Xu *et al.*, 2009 ▶; Aramini *et al.*, 2008 ▶). Therefore, we docked the tripeptide l-­Ala-γ-d-Glu-l-DAP into the active site of BcYkfC in order to deduce a likely mode of interaction and to assess the effects of the differences (Fig. 6 ▶ *a*). The C^δ^ carboxyl group of γ-d-Glu is displaced owing to the oxidation of Cys238 in the crystal structure. In the modeled structure, Tyr226 OH interacts with the carboxyl group of γ-­d-Glu (Fig. 6 ▶ *b*). This docked structure places the C^δ^ atom close to Cys238 SH (distance ∼3.6 Å) and could represent the chemically productive conformation. The electrostatic surface of the binding site is highly complementary to that of the substrate, indicating that the polar and charged interactions are likely to be important for substrate recognition. The basic His290 and Arg306 residues could interact with carboxyl groups. It has previously been reported that the catalytic efficiency of *B. subtilis* YkfC for l-Ala-γ-d-Glu-Lys is significantly less than that for tetrapeptides or pentapeptides (Schmidt *et al.*, 2001 ▶). This observation could be explained by the unfavorable basic environment in the S′ site. Two residues (Arg306/Glu308) at the BcYkfC S1′ site are substituted by Lys/Gly in *B. subtilis* YkfC (Fig. 5 ▶), resulting in a subsite with a positive charge but no negative charge that would be less likely to accommodate the positively charged Lys of l-­Ala-γ-d-Glu-Lys (Fig. 6 ▶ *a*).

### 3.8. Recognition of γ-d-Glu by NlpC/P60 cysteine peptidases

NlpC/P60 cysteine peptidases are ubiquitous in bacteria, with ∼5000 NlpC/P60 domains in the current Pfam database when metagenomics data are included (Bateman *et al.*, 2004 ▶). However, only a few of these domains have been biochemically characterized. In the light of the much clearer picture that we now have of substrate binding in BcYkfC, we examined the sequence conservation of NlpC/P60-family proteins around the active site in order to obtain further insights into substrate specificity across the entire family.

The nonredundant (nr) database of protein sequences was searched using the *PSI-BLAST* program (Altschul *et al.*, 1997 ▶) with the NlpC/P60 domain of BcYkfC as a search probe. We extracted a total of 2599 protein sequences using the criteria of an *E* value of >0.02 and an alignment length of >70 residues from 3985 total hits, representing a significant subset of NlpC/P60 proteins. Among these proteins, only ∼1% do not possess the conserved Cys/His dyad and an additional 9% (with catalytic dyad intact) do not contain the conserved tyrosine that is believed to be essential for catalysis. These proteins are likely to have the NlpC/P60 fold, but either have lost cysteine peptidase activity or are remote homologs that hydrolyze different substrates (*e.g.* the CHAP family; Anantharaman & Aravind, 2003 ▶; Bateman & Rawlings, 2003 ▶; Rigden *et al.*, 2003 ▶). The sequence-conservation pattern of active-site residues of the remaining 2277 proteins (average sequence identity of 19%) is shown in Fig. 6 ▶(*c*). The S sites (S2 and S1) are significantly more conserved than the S′ sites in these proteins. The most conserved site is S1, which is tailored to recognize γ-d-Glu (Fig. 6 ▶ *b*). The three most highly conserved residues (Asp237, Ser239 and Tyr226), aside from the catalytic dyad, are involved in hydrogen bonding with γ-d-Glu (Fig. 6 ▶ *b*). At position 257, residues with small side chains (Ala/Ser/Thr) are observed, creating a cavity for the carboxyl group of γ-d-Glu. Trp228 contributes to both the S1 and S2 sites and a conserved hydrophobic residue is usually found at this position, which may facilitate interaction with the substrate Ala C^β^. Therefore, the conservation of active-site residues indicates that a significant percentage of the NlpC/P60 family is likely to be specific for γ-d-Glu. More than 1300 proteins which contain the strictly conserved Trp228, Asp237, Ser239 and Tyr226 residues (and catalytic triads) are most likely to bind *X*-l-Ala-γ-d-Glu moieties (where *X* is H or other moieties).

### 3.9. Structural comparison to the cyanobacterial NlpC/P60 l-Ala-γ-d-Glu endopeptidases

**Figure 7:**
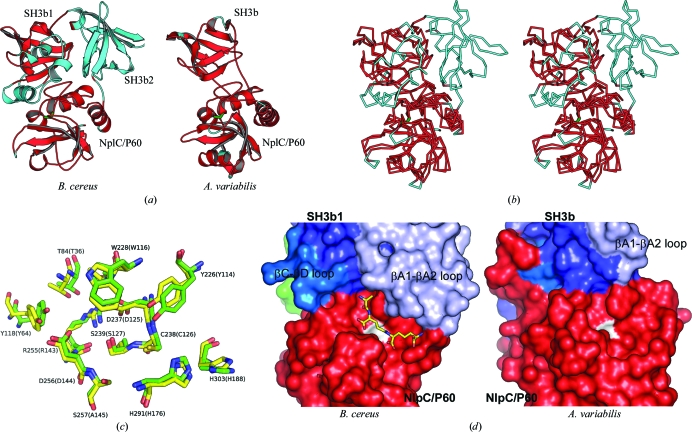
Structural comparisons between BcYkfC and the cyanobacterial NlpC/P60 endopeptidase AvPCP. (*a*) BcYkfC and AvPCP (PDB code 2hbw; Xu *et al.*, 2009 ▶) are shown with the same orientation of their common SH3b1 and NlpC/P60 domains. The structurally equivalent residues in each are shown in red. (*b*) Stereoview of the C^α^ traces of the two proteins shown in (*a*) [the coloring is the same as in (*a*)]. (*c*) The S binding sites of the two proteins are nearly identical. The corresponding residues of AvPCP are labeled in parentheses. (*d*) Comparison of the active-site cavities and their environments. The catalytic cysteine is shown in white. A stick model of a docked murein tripeptide is shown in the active site of BcYkfC.

We have previously determined the crystal structures of two closely related NlpC/P60 l-Ala-γ-d-Glu endopeptidases from the cyanobacteria *A. variabilis* (AvPCP) and *N. punctiforme* (NpPCP). These enzymes contain an N-terminal SH3b domain and a C-terminal NlpC/P60 catalytic domain (Xu *et al.*, 2009 ▶). Surprisingly, the structures of the cyanobacterial enzymes are essentially a substructure of YkfC: the two domains of the cyanobacterial enzymes are structurally equivalent to the first and third domains of BcYkfC (Figs. 5 ▶, 7 ▶ *a* and 7 ▶ *b*). The full-length AvPCP can be superimposed onto BcYkfC with an r.m.s.d. of 2.3 Å and 29% sequence identity for 193 aligned C^α^ atoms (Table 2 ▶). Thus, the individual SH3b and NlpC/P60 domains, as well as their relative arrangements, are highly similarly in AvPCP and BcYkfC. Furthermore, the residues in the S2 and S1 pockets are nearly identical (except for S257A; Fig. 7 ▶ *c*). The striking similarity between the cyanobacterial enzymes and YkfC was not previously detected by sequence analysis owing to the presence of two insertions: the entire SH3b2 domain and the α1–α3 region of SH3b1.

The βC–βD loop and the βA1–βA2 loop of SH3b1 are longer compared with SH3b of AvPCP. The βC–βD loop of YkfC is located upstream of the S2 site. The βA1–βA2 loop of YkfC contains three helices (α1–α3) and is located on the S′ side of the binding groove in the catalytic domain. This insertion packs against the surface of the NlpC/P60 domain and contributes to stabilizing the interface between the SH3b1 and NlpC/P60 domains. Furthermore, it is also defines the shape of the S′ binding site, which appears to be more restricted for BcYkfC than for AvPCP (Fig. 7 ▶ *d*). Without these insertions to stabilize this domain interface, AvPCP uses a different strategy to achieve a similar purpose: a longer C-terminal loop in NlpC/P60 extends to interact with the SH3b domain.

We previously proposed that the cyanobacterial enzymes have the same substrate requirement as DPP VI based on modeling studies (Xu *et al.*, 2009 ▶). The crystal structure of YkfC reported here further supports DPP VI, YkfC and the cyanobacterial enzymes belonging to a subset of NlpC/P60 γ-d-Glu-DAP endopeptidases whose sub­strates possess a free l-Ala at the N-terminus. The SH3b domain helps define the S2 binding site (Tyr118 in BcYkfC and Tyr64 in AvPCP). Additionally, it sterically hinders the docking of a large moiety beyond the S2 site and directly contributes to specificity. This role constitutes a new function for SH3b domains.

### 3.10. Identification and distribution of YkfC homologs

BcYkfC and cyanobacterial γ-d-Glu-DAP endopeptidases have the same specificity. Other NlpC/P60 enzymes with similar properties could potentially be detected by analyzing sequence similarities in both the SH3b and NlpC/P60 domains. However, owing to the pre­sence of long insertions/deletions and highly divergent sequences in the SH3b domains, it is often difficult to detect similar enzymes using sequence searches and the NlpC/P60 region tends to dominate the hits. We examined an alternate method by examining specific residues in the NlpC/P60 domain only. An acidic residue (Asp256) is essential for YkfC specificity by neutralizing the positive charge on the free amine of l-Ala. Among the 2599 sequences obtained above, 282 proteins were identified that contained an aspartate equivalent to Asp256 and a conserved catalytic dyad. In 239 of the 282 proteins (85%), the conserved tyrosine was maintained in their SH3b domains (Tyr118 of BcYkfC), while this tyrosine was not conserved in enzymes that lacked Asp256. Thus, the presence of an aspartate residue at position 256 and a conserved tyrosine in SH3b is highly correlated (Fig. 6 ▶ *d*). Interestingly, NlpC/P60 domains with the con­served aspartate are also found in a few single-domain or multiple-domain proteins that do not contain any detectable SH3b domain. The biochemical properties of these proteins are currently unknown, but they could represent novel variants of a YkfC-type enzyme.

Nevertheless, the 239 proteins above with the conserved aspartate (Asp256) and tyrosine (Tyr118) are most likely to define a YkfC-like subfamily of NlpC/P60 proteins. These NlpC/P60 enzymes are predominately distributed in four phyla of bacteria: bacteroidetes, cyanobacteria, firmicutes and proteobacteria (α-proteobacteria). They include all currently known enzymes with the same requirement for a free N-terminal l-Ala, *i.e.* DPP VI, YkfC and AvPCP/NpPCP. The cyanobacterial enzymes contain one SH3b domain, while the homologs in other bacteria contain two SH3b domains. As expected, the SH3b domains are highly divergent. The βD strand, in which the conserved tyrosine is located, is more conserved (Fig. 6 ▶ *d*). This strand is also responsible for the interface with the catalytic domain. BcYkfC is a member of a cluster of highly conserved homologs that are present in closely related species such as *B. anthracis*, *B. thuringiensis* and *B. weihenstephanensis* (sequence identity of ≥95%). All members of this group contain signal peptides. Signal peptides are also present in some members of the bacteroidetes group, but not in proteobacterial and cyanobacterial homologs.

It is thus likely that the YkfC subfamily of highly specialized enzymes has evolved from a common ancestor, which was likely to have been a P60-like general-purpose enzyme with two or more SH3b domains connected to a C-terminal NlpC/P60 domain. SH3b domains may have lost the function of targeting domains and over time the nonessential SH3b domain would have been lost, as seen in the cyanobacterial enzymes.

## 4. Conclusions

The structure of BcYkfC in complex with l-Ala-γ-d-Glu is the first structural representative of an NlpC/P60 enzyme with a bound ligand. This structure allowed us to identify the determinants of substrate specificity, which further led to the classification of a subfamily of highly specialized NlpC/P60 enzymes. The studies here also provide structural and functional insights into the NlpC/P60 family of enzymes. Additional information about BcYkfC is available from TOPSAN (Krishna *et al.*, 2010 ▶) at http://www.topsan.org/explore?PDBid=3h41.

## Supplementary Material

PDB reference: YkfC–l-Ala-γ-d-Glu complex, 3h41

## Figures and Tables

**Table 1:** Data-collection, phasing and refinement statistics (PDB code 3h41) Values in parentheses are for the highest resolution shell. The high-resolution cutoff was chosen such that the mean *I*/σ(*I*) in the highest resolution shell was around 2.

	λ_1_ MADSe, peak	λ_2_ MADSe, remote	λ_3_ MADSe, inflection
Space group	*C*2		
Unit-cell parameters (Å, °)	*a* = 95.2, *b* = 59.4, *c* = 61.3, β = 103.3		
Data collection			
Wavelength (Å)	0.9786	0.9184	0.9799
Resolution range (Å)	41.5–1.79 (1.88–1.79)	41.5–1.84 (1.94–1.84)	41.5–1.86 (1.96–1.86)
No. of observations	116822	107729	104295
No. of unique reflections	31085	28629	27652
Completeness (%)	98.0 (95.1)	98.6 (98.1)	98.5 (97.9)
Mean *I*/σ(*I*)	11.3 (2.1)	12.5 (2.8)	13.1 (2.8)
*R*_merge_ on *I*† (%)	11.1 (71)	10.0 (54)	10.0 (56)
MAD phasing			
Resolution	41.5–1.79		
No. of Se sites	5		
Mean figure of merit	0.36		
Model and refinement statistics			
Resolution range (Å)	41.5–1.79		
No. of reflections (total)	31083		
No. of reflections (test)	1564		
Completeness (%)	98.0		
Data set used in refinement	λ_1_ MADSe		
Cutoff criterion	|*F*| > 0		
*R*_cryst_‡ (%)	16.3		
*R*_free_§ (%)	19.7		
Stereochemical parameters			
Restraints (r.m.s.d. observed)			
Bond lengths (Å)	0.015		
Bond angles (°)	1.50		
Average isotropic *B* value (Å^2^)	19.9		
ESU¶ based on *R*_free_ (Å)	0.11		
Protein residues/atoms	305/2463		
Waters/peptide/other ligands	265/1/7		
*MolProbity* statistics			
All-atom clash score	5.07		
Ramachandran favored (%)	97.7		
No. of Ramachandran outliers	1		
No. of rotamer outliers	1		

† *R*_merge_ = 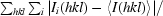 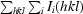.

‡ *R*_cryst_ = 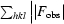 − 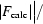 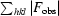, where *F*_calc_ and *F*_obs_ are the calculated and observed structure-factor amplitudes, respectively.

§ *R*_free_ is the same as *R*_cryst_ but for 5.0% of the total reflections chosen at random and omitted from refinement.

¶ Estimated standard uncertainty in atomic coordinates.

**Table 2:** Structural comparisons of BcYkfC and other bacterial proteins that share at least one common domain The alignment was performed by the *DALI* structural comparison server using full-length BcYkfC and individual domains (SH3b1 and NlpC/P60) as search probes. For proteins with multiple SH3-like domains (PDB codes 1m9s and 1xov) or multiple chains, only the best match is shown.

Aligned substructure(s)	PDB code	Reference	R.m.s.d. (Å)	Aligned length	No. of residues†	*Z* score	Sequence identity (%)	Comments
SH3b + NlpC/P60	2fg0	Xu *et al.* (2009 ▶)	2.1	193	221	21.3	26	Cyanobacterial NpPCP
	2hbw	Xu *et al.* (2009 ▶)	2.3	193	220	21.2	29	Cyanobacterial AvPCP
SH3b	1m9s	Marino *et al.* (2002 ▶)	1.9	60	523	6.0	12	TD‡ of internalin B
	1r77	Lu *et al.* (2006 ▶)	2.3	57	103	4.7	12	TD‡ of PG hydrolase ALE-1
	1xov	Korndorfer *et al.* (2006 ▶)	3.0	51	317	3.5	10	TD‡ of endolysin PlyPSA
	2akk	Srisailam *et al.* (2006 ▶)	1.8	44	74	2.7	7	PhnA-like protein
	1x27	Nasertorabi *et al.* (2006 ▶)	1.9	54	164	5.7	13	Eukaryotic SH3
NlpC/P60	2jyx	Aramini *et al.* (2008 ▶)	1.9	119	129	17.8	29	*E. coli* lipoprotein Spr
	2p1g	NYSGXRC§	2.5	110	230	9.6	18	Uncharacterized
	2ioa	Pai *et al.* (2006 ▶)	3.2	106	589	7.3	9	CHAP domain of GspS¶

† Number of residues present in the model used for comparison.

‡ Targeting domain.

§ New York SGX Research Center for Structural Genomics (unpublished work).

¶ Glutathionylspermidine synthetase/amidase.
